# Derivatives of Pyridazine with Phenoxazine and 9,9-Dimethyl-9,10-dihydroacridine Donor Moieties Exhibiting Thermally Activated Delayed Fluorescence

**DOI:** 10.3390/ma16031294

**Published:** 2023-02-02

**Authors:** Levani Skhirtladze, Oleksandr Bezvikonnyi, Rasa Keruckienė, Lukas Dvylys, Malek Mahmoudi, Linas Labanauskas, Azhar Ariffin, Juozas V. Grazulevicius

**Affiliations:** 1 Department of Polymer Chemistry and Technology, Faculty of Chemical Technology, Kaunas University of Technology, LT-51423 Kaunas, Lithuania; 2 Department of Physics, Faculty of Mathematics and Natural Science, Kaunas University of Technology, LT-51369 Kaunas, Lithuania; 3 Center for Physical Sciences & Technology, Department of Organic Chemistry, LT-10257 Vilnius, Lithuania; 4 Department of Chemistry, Faculty of Science, Universiti Malaya, Kuala Lumpur 50603, Malaysia

**Keywords:** pyridazine, 9,9-dimethyl-9,10-dihydroacridine, phenoxazine, delayed fluorescence

## Abstract

Two compounds based on pyridazine as the acceptor core and 9,9-dimethyl-9,10-dihydroacridine or phenoxazine donor moieties were designed and synthesized by Buchwald–Hartwig cross-coupling reaction. The electronic, photophysical, and electrochemical properties of the compounds were studied by ultraviolet-visible spectroscopy (UV-vis), photoluminescence spectrometry, differential scanning calorimetry, thermogravimetric analysis, and cyclic voltammetry. The compounds are characterized by high thermal stabilities. Their 5% weight loss temperatures are 314 and 336 °C. Complete weight loss of both pyridazine-based compounds was detected by TGA, indicating sublimation. The derivative of pyridazine and 9,9-dimethyl-9,10-dihydroacridine is capable of glass formation. Its glass transition temperature is 80 °C. The geometries and electronic characteristics of the compounds were substantiated using density functional theory (DFT). The compounds exhibited emission from the intramolecular charge transfer state manifested by positive solvatochromism. The emission in the range of 534–609 nm of the toluene solutions of the compounds is thermally activated delayed fluorescence with lifetimes of 93 and 143 ns, respectively.

## 1. Introduction

**Figure 1:**
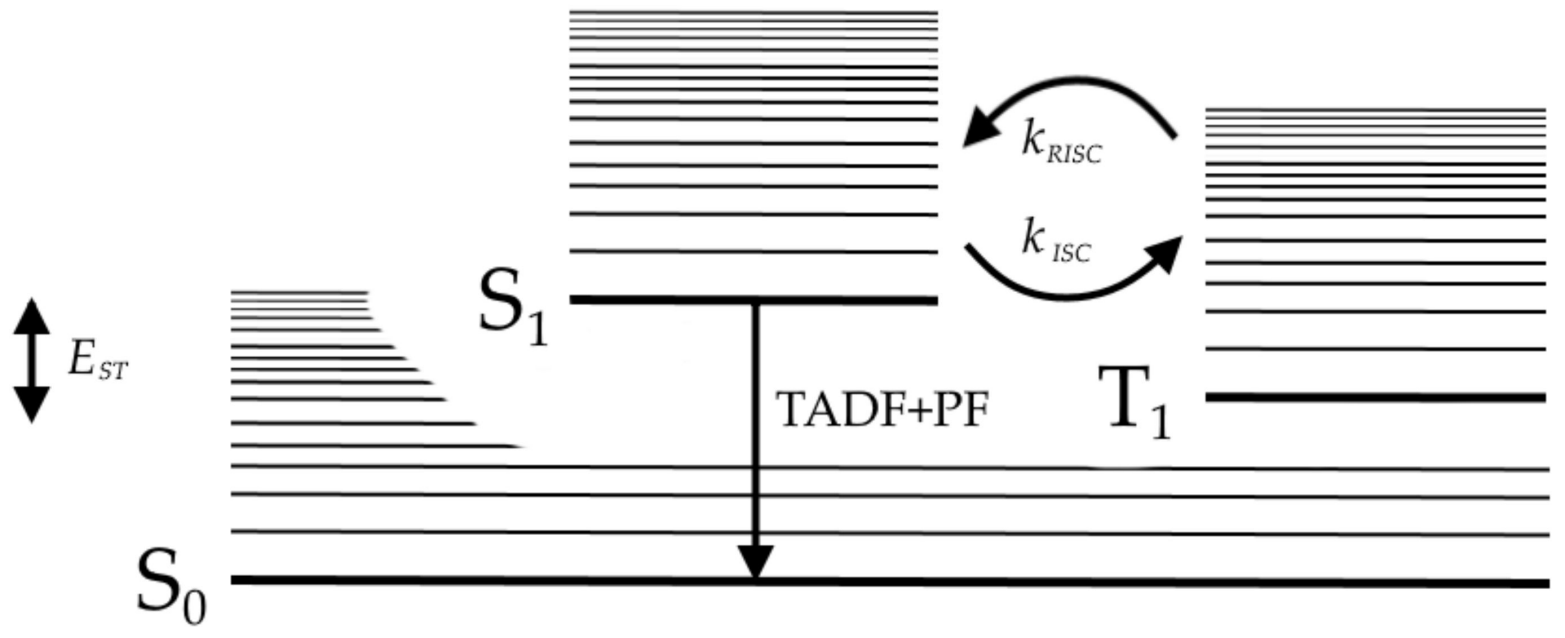
Simplified Jablonski diagram for TADF emitters.

Thermally activated delayed fluorescence (TADF) has emerged as a promising photophysical mechanism useful in the development of efficient organic light emitting diodes (OLEDs) [1,2,3,4]. TADF is a phenomenon that relies on the upconversion of triplet excitons to singlet excited states by means of reverse intersystem crossing (RISC) due to the thermal motion of atoms [5,6,7] (Figure 1).

OLEDs based on TADF are an alternative type of OLED to phosphorescent OLEDs (PhOLEDs) [8]. The radiative utilization of triplet electronic excitation energy in the case of PhOLEDs is due to the exciton-spin-orbit-photon interaction relying on the largest atomic number of atoms incorporated in the structure [8]. Therefore, the phosphorescent emitters of OLEDs commonly have noble metal atoms such as Ir or Pt attached to the organic moieties. TADF is of great interest in this regard, as it allows the utilization of triplet energy without the use of expensive materials. Efficient RISC can be reached by combining electron-accepting and electron-donating moieties in a single molecule and constraining their dihedral angles in such a way that it minimizes the overlap of HOMO and LUMO [9,10,11]. Electron-withdrawing moieties containing cyano groups or azoheterocycles have been used most extensively for the design of TADF emitters [8,9,10,11,12,13,14,15,16]. The use of 9-[4-(4-(4-(4,6-diphenyl-1,3,5-triazin-2-yl) phenyl]-N, N, N0, N0-tetraphenyl-9H-carbazole-3,6-diamine (DACT-II), which has a triazine electron-acceptor (A) moiety, allowed the design of OLEDs with internal quantum efficiency of 100% [17]. 1,2,3,5-Tetrakis(carbazol-9-yl)-4,6-dicyanobenzene (4CzIPN), with dicyanobenzene as the electron acceptor, has shown excellent redox window, good stability, and excellent TADF characteristics [18].

Aromatic azaheterocycles are among the most electron-deficient aromatic heterocycles [19,20]. Triazine, pyridine, and naphthyridine contain strongly electronegative nitrogen atoms in their molecular skeletons. Therefore, they display an intrinsic electron-deficient character, enabling them to be used in the design of compounds for a wide range of applications, such as pH sensing, optoelectronics, nonlinear optical materials, and pharmaceuticals [21,22,23,24]. Reports on the synthesis of the studied pyridazine derivatives for optoelectronic and related applications are still scarce. They were reported as host materials for OLEDs and liquid-crystalline materials, and as phosphorescent iridium complexes [25,26,27,28]. A series of pyridazine-based TADF emitters have been described, where the combination of the pyridazine moiety with phenoxazine resulted in an emitter with a photoluminescence quantum efficiency of 10.9% with an estimated high RISC rate k_RISC_ of 3.9 × 10^6^ s^−1^ and a small singlet–triplet splitting value of 86 meV [29]. The OLED based on this emitter demonstrated external quantum efficiency of over 5.8%, confirming triplet utilization in the device via TADF [29]. Pyridazine is not only more electron-accepting, but also more polar than pyridine, which is beneficial for its use as an electron acceptor in the design of D-A-type materials [28]. In this work, the synthesis and properties of two derivatives of pyridazine bearing phenoxazine and 9,9-dimethyl-9,10-dihydroacridine electron-donating moieties are reported. The interesting structure–properties relationship was disclosed by studies of thermal, photophysical, and electrochemical properties, as well as by quantum chemistry studies. Investigation of the photophysical properties of the toluene solutions and films of the compounds confirmed that TADF showed lifetimes of 93 and 143 ns, corresponding to the toluene solutions of the compounds having phenoxazine and 9,9-dimethyl-9,10-dihydroacridine, respectively.

## 2. Results and Discussion

### 2.1. Synthesis and Thermal Properties

**Scheme 1:**
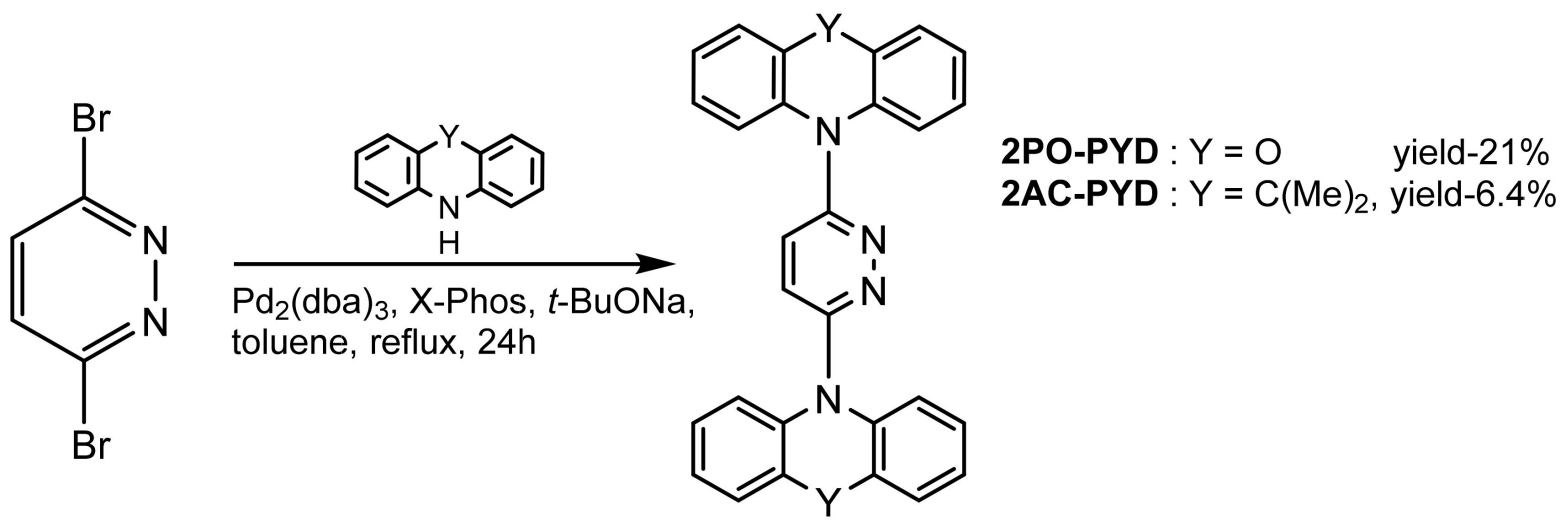
Synthesis of 2,5-disubstituted pyridazine derivatives.

The synthesis of the derivatives of 2,5-disubstituted-pyridazine is shown in Scheme 1. For the synthesis of the target compounds, Buchwald–Hartwig coupling reactions of brominated pyridazine with phenoxazine or 9,9-dimethyl-9,10-dihydroacridine were carried out in the presence of tris(dibenzylideneacetone)dipalladium(0) as the metal catalyst and X-Phos as the ligand. The reactions produced donor–acceptor–donor (D-A-D) derivatives 2PO-PYD and 2AC-PYD in yields of 21.8% and 6.4%, respectively. The target compounds were purified by column chromatography. The structures of the synthesized compounds were confirmed by ^1^H, ^13^C NMR, and FT-IR spectroscopies, as well as mass spectrometry. They were found to be soluble in common organic solvents. Characteristics of the compounds 2PO-PYD and 2AC-PYD can be found in the Supplementary Materials File.

Morphological transitions and thermal stabilities of pyridazine-based compounds (2PO-PYD and 2AC-PYD) were investigated by differential scanning calorimetry (DSC) and thermogravimetric analysis (TGA) (Figure S5). Their thermal characteristics are shown in Table 1.

Both target compounds were obtained after the synthesis and purification as crystalline substances. Endothermic melting signals were observed in the first heating scans of the DSC measurements (Figure S5a,b). Crystallization and subsequent melting signals were observed during the cooling and second heating scans. The glass transition of compound 2AC-PYD was detected during the second heating scan, indicating the possibility of transformation into a solid amorphous state (molecular glass). During the TGA measurements, complete weight loss of both pyridazine-based compounds was detected, indicating sublimation rather than thermal degradation of the samples.

### 2.2. Theoretical Calculations and Electrochemical Properties

**Figure 2:**
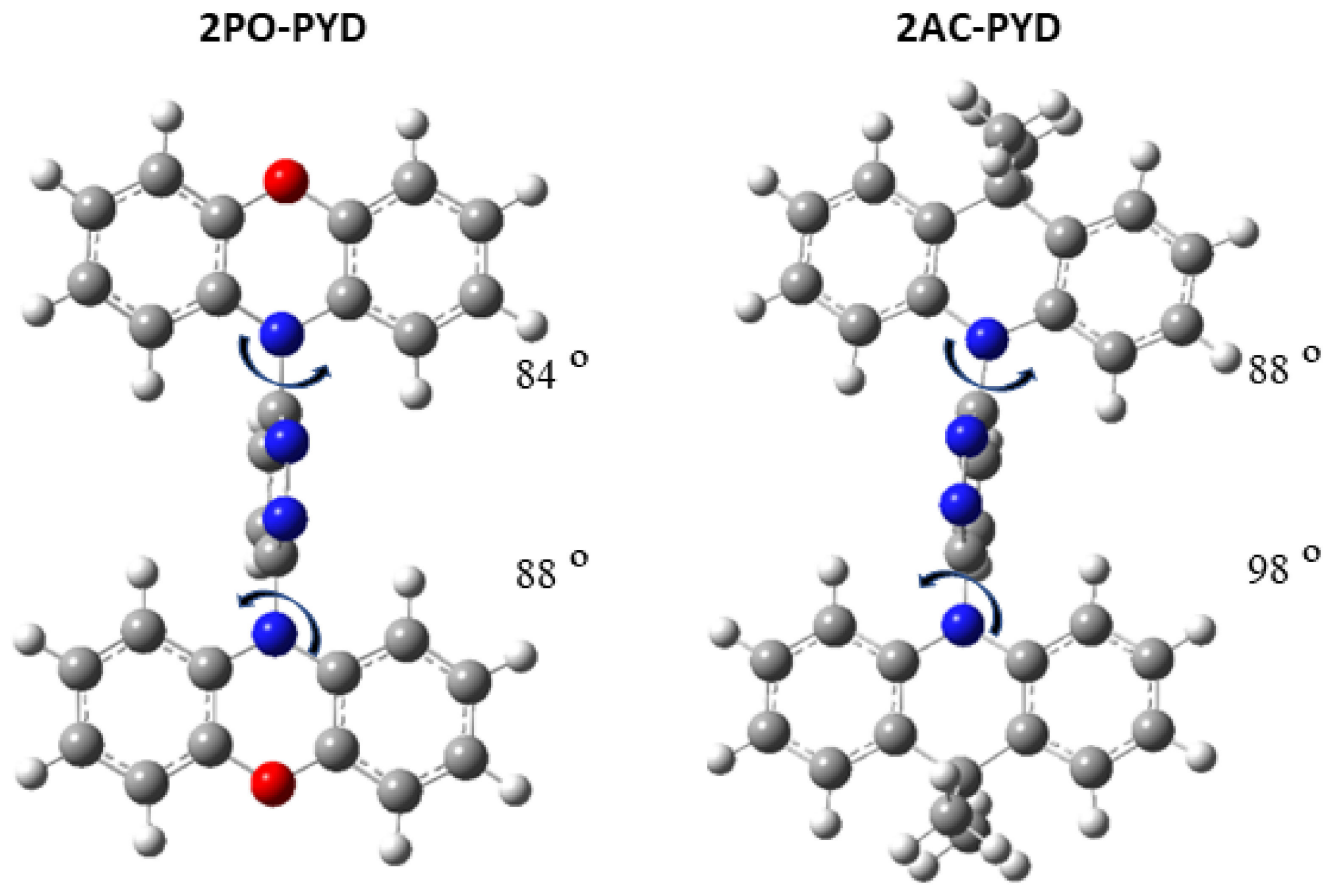
Optimized ground-state geometries in vacuum at the B3LYP/6-31++G level of theory of pyridazine derivatives **2PO-PYD** and **2AC-PYD** (grey color: carbon; blue: nitrogen; red: oxygen; white: hydrogen).

The geometries and electronic structures of the target molecules were analyzed using DFT calculations at the B3LYP/6-31++G theoretical level. The ground-state geometries were optimized by using the B3LYP (Becke three parameters hybrid functional with Lee-Yang-Perdew correlation) [30] functional at 6-31G (d, p) level in vacuum with the Gaussian program [31]. To evaluate the electronic transitions, the geometries of the pyridazine derivatives **2PO-PYD** and **2AC-PYD** were evaluated (Figure 2). As the acceptor and donor moieties are directly linked, the values of the dihedral angles are crucial for intramolecular charge transfer (ICT). In the optimized ground-state geometries, the D and A fragments are almost perpendicularly orientated, as their dihedral angle values are close to 90°. Such large dihedral angle values are expected to lead to minimal conjugation of the D-A fragments.

**Figure 3:**
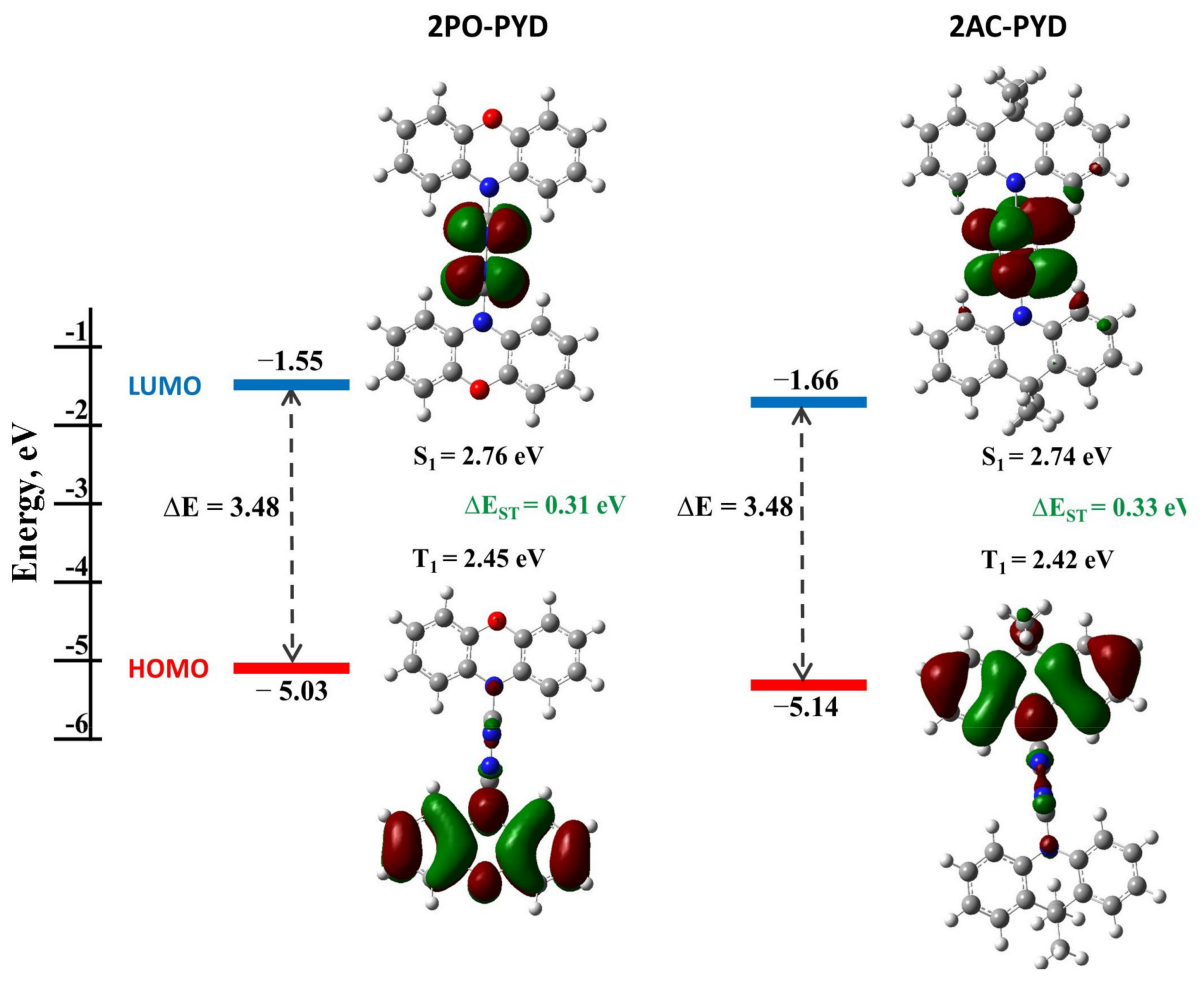
DFT calculated HOMO and LUMO energies, as well as HOMO and LUMO topologies (isovalue of 0.02), of compounds **2PO-PYD** and **2AC-PYD**.

The calculated HOMOs and LUMOs are presented in Figure 3. The electronic structures of both pyridazine-based compounds are similar. The HOMOs are situated on the electron-donating fragments of phenoxazine and 9,9-dimethyl-9,10-dihydroacridine, with a small electron density residing on the pyridazine fragment, whereas the LUMO is situated solely on the acceptor fragment. The calculated HOMO levels confirm the slightly stronger electron-donating strength of phenoxazine compared to that of the 9,9-dimethyl-9,10-dihydroacridine moiety. The levels of LUMO are in good agreement with the LUMO values of previously reported pyridazine-based host compounds [29].

In the optimized excited-state geometry, the dihedral angles remain perpendicular. TD-DFT calculations predicted that the dominant S1 transition is CT in nature (H→L). The calculated values of the energy gaps between the singlet and triplet states (ΔE_ST_) are ca. 0.3 eV (Figure 3), and are in line with the slightly stronger electron-donating nature of the phenoxazine moiety. Such small energy gaps make these pyridazine derivatives promising as TADF emitters.

**Figure 4:**
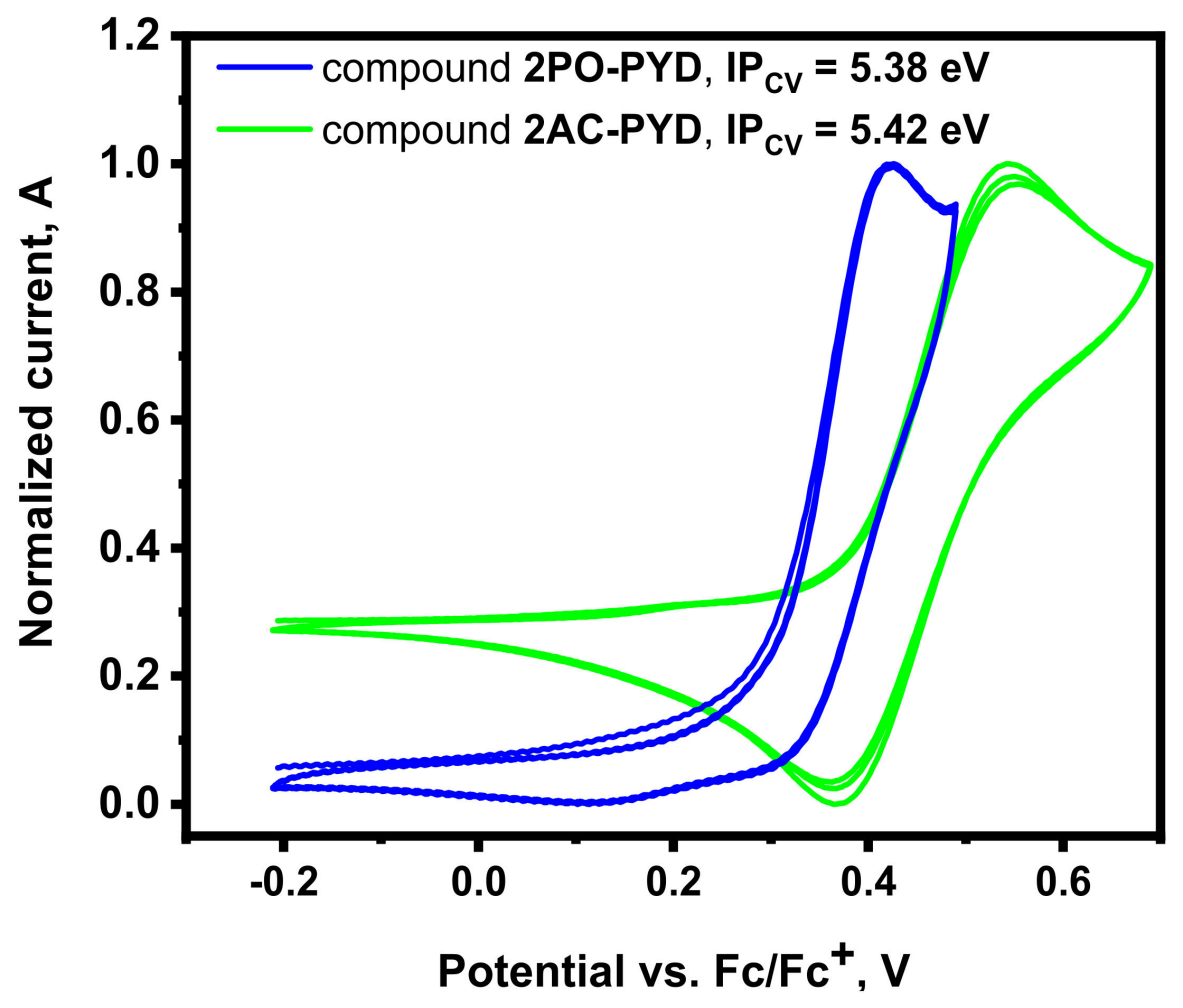
Cyclic voltammograms of compounds **2PO-PYD** and **2AC-PYD**.

Cyclic voltammetry was used to investigate the electrochemical properties of pyridazine-based compounds **2PO-PYD** and **2AC-PYD**. **2AC-PYD** showed reversible oxidation during repeated scans, whereas the pyridazine-based compound **2PO-PYD** showed quasi-reversible oxidation. Ionization potentials estimated by the cyclic voltammetry (IP_CV_) values were estimated from oxidation onset potentials against ferrocene (Eox onset vs. Fc). The voltammograms are shown in Figure 4.

The IP_CV_ values were found to be only slightly dependent on the strength of the donor moieties attached. The phenoxazine-containing compound (**2PO-PYD**) showed slightly higher IP_CV_ than the derivative of 9,9-dimethyl-9,10-dihydroacridine (**2AC-PYD**). Despite the small differences, overall, the experimentally determined characteristics correlate well with the theoretically calculated ones.

### 2.3. Photophysical Properties

**Figure 5:**
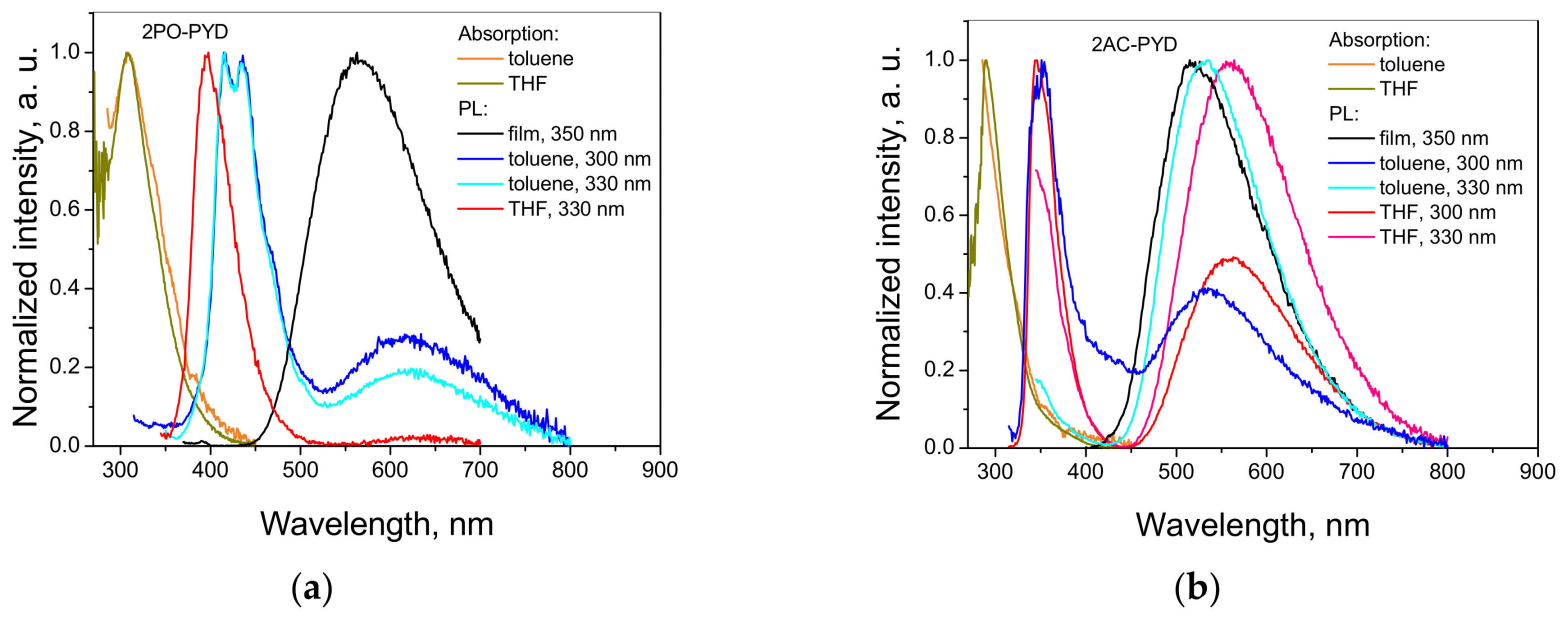

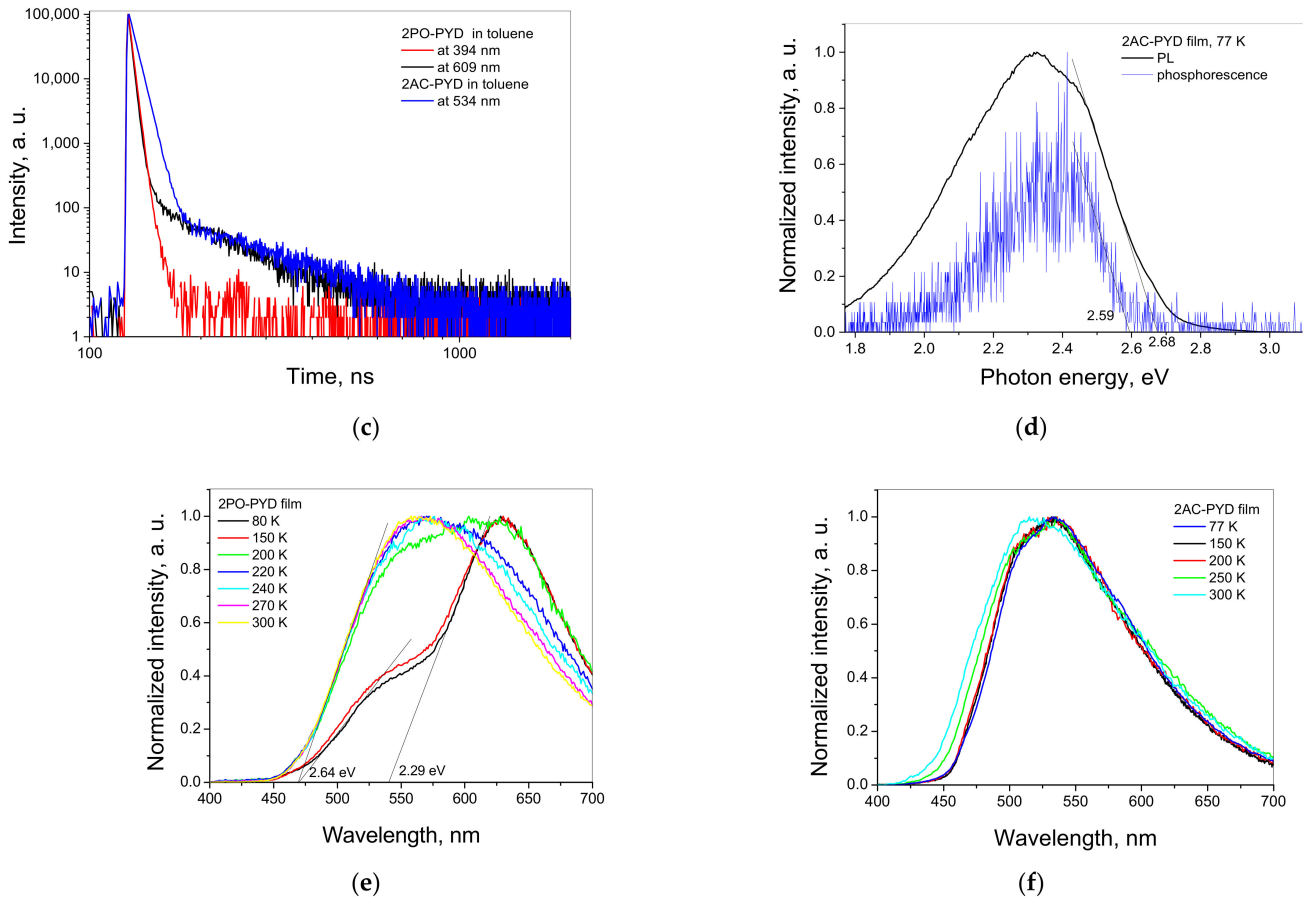
Absorption and PL spectra of toluene, THF solutions, and films of **2PO-PYD** (**a**) and **2AC-PYD** (**b**). PL decay curves of deoxygenated toluene solutions (**c**). PL and phosphorescence spectra of the film of **2AC-PYD** recorded at liquid nitrogen temperature (**d**). PL spectra of the films of **2PO-PYD** (**e**) and **2AC-PYD** (**f**) recorded in inert atmosphere at different temperatures.

The absorption and emission spectra of the solutions of **2PO-PYD** and **2AC-PYD** are presented in Figure 5a,b, respectively, and in Table 2.

The absorption spectra are characterized by the prominent band at 308 nm for the solutions of **2PO-PYD** and 288 nm for those of **2AC-PYD**. The positions of the absorption peaks almost did not change when changing the non-polar toluene solvent with the polar THF. DFT predicts that this band originates from the locally excited (LE) state of donor fragments (Figure S6). The very low oscillator strength of 0.002 of the transition of the ICT state (S_0_→S_1_) is apparently the reason for the lack of this absorption band in the experimental UV spectra.

The different photoluminescence (PL) spectra of the solutions recorded at the different excitation wavelengths showed the presence of two distinguished emission bands in the UV and green spectral regions. The spectral data presented in Table 2 provide an opportunity to track the influence of polarity of the medium on photophysical characteristics of the compounds. Toluene is a non-polar solvent, while THF is a moderately polar one. The investigated compounds are designed to have a donor–acceptor–donor structure; thus, the dipole moment must be affected by the polarity of the solvents, exhibiting solvatochromism. The PL band in the UV region covering the range of ca. 400–450 nm is related to the local excited (LE) state emission of the donor. It is not characterized by the positive solvatochromism caused by the increase in polarity of the solvent. The low-energy emission band at ca. 500–625 nm exhibited a spectral redshift with the increase of the solvent polarity, pointing to the intramolecular charge transfer (ICT) state. Different excitation wavelengths alter the spectral distribution insignificantly. This observation is in full accordance with Dr. Michael Kasha’s rule excluding the possibility of excitation of specific optical centers affecting the analysis of the experimental data. The ICT peak wavelengths of the spectra of neat films of the compounds correspond to the peak positions of the ICT band of respective toluene solutions. This demonstrates the absence of the effect of intermolecular interactions in solid state, polarity, or aggregation-related phenomena on the ICT of the compounds. The emission of 9,9-dimethyl-9,10-dihydroacridin-based **2AC-PYD** is expectedly blue-shifted with respect to that of **2PO-PYD** with a stronger donating unit of phenoxazine. The PL quantum yields of the deoxygenated toluene solutions *Φ* of both **2PO-PYD** and **2AC-PYD** did not reach 1%. The deoxygenation of the solutions and of the solid films of the compounds did not lead to the change of the photoluminescence spectra, highlighting the ICT nature of the respective band at ca. 500–625 nm and the absence/weakness of the room-temperature phosphorescence.

The PL decay curves of the deoxygenated toluene solutions of **2PO-PYD** and **2AC-PYD** were recorded at the corresponding PL peak wavelengths (Figure 5c). The major data of PL decays are collected in Table 3 and Figure S7. The multiexponential fitting exhibited that the lifetime of the **2PO-PYD** LE band totally correlates with the lifetime of the prompt fluorescent component of the PL decay curve recorded at 621 nm. At the same time, the ICT bands of the solutions of **2PO-PYD** and **2AC-PYD** exhibited emission lifetimes of 93 and 143 ns, respectively. Such lifetimes are associated with the so-called fast delayed emission, i.e., TADF [32].

PL spectra of the films of **2PO-PYD** and **2AC-PYD** were taken at different temperatures to decisively confirm the absence of room-temperature phosphorescence. The energy levels of the first excited singlet and triplet states were estimated from the onsets of the respective prompt fluorescent and phosphorescent bands (Figure 5d,e). The phosphorescence component of the emission disappeared while heating, leaving only the ICT band (Figure 5e,f). The presumption about the TADF phenomenon is inconsistent with the obtained *ΔE_ST_* of 0.35 eV (Figure 5e) between the first singlet and triplet excited states of **2PO-PYD**, even though it is predicted based on the results of the theoretical analysis (Figure 3). In this case, the RISC relies heavily on a spin–orbit coupling of energetically close ^3^CT and ^3^LE states. The possibility of spin being flipped in the organic material without insertion of heavy atoms into the structure is realized because *k_RISC_* is deeply dependent on the triplet excitonic Bohr radius, which enhances *k_RISC_* by several orders of magnitude more than the rate constant of ISC (*k_ISC_*) (Table 3) [11]. The energy level of 2.29 eV is attributed to ^3^CT of **2PO-PYD**, similar to previously reported phenoxazine-based TADF compounds [28,29,31]. The presence of ^3^LE is anticipated with an energy level close to that of ^1^CT state, i.e., of ca. 2.64 eV (Figure 5e), facilitating TADF [32,33]. The low *Φ* makes it difficult to detect the ^3^LE experimentally. As expected for **2AC-PYD** containing the 9,9-dimethyl-9,10-dihydroacridine donor moiety [28,29,32,34,35], the first triplet excited state with the energy of 2.59 eV is close to that of ^1^CT of 2.68 eV, which is beneficial for TADF. The values of *k_RISC_* and *k_ISC_* were evaluated from the fitting data of the PL decay curves using equations $k_{RISC}=\frac{(\Phi_{PF}+\Phi_{DF})\Phi_{RISC}}{\tau_{DF}\Phi_{PF}}$, $k_{ISC}=\frac{\Phi_{DF}}{\tau_{PF}\left(\Phi_{PF}+\Phi_{DF}\right)}$, where *Φ_PF_*, *Φ_DF_*, and *Φ_RISC_* are the quantum yields of prompt, delayed fluorescence, and RISC, respectively [36]. Using the formula $\Phi =\Phi_{PF}+\Phi_{DF}=\frac{\Phi_{PF}}{1-\Phi_{ISC}\Phi_{RISC}}$ and knowing that the yield of ISC *Φ_ISC_* cannot exceed the electronic excitation energy not utilized in prompt fluorescence, *k_RISC_* can be estimated by the formula $k_{RISC}=\frac{\Phi_{DF}}{\tau_{DF}\Phi_{PF}(1-\Phi_{PF})}$ [37]. The data obtained are collected in Table 3. The *k_RISC_* value for 2PO-PYD reached almost 10^6^ s^−1^, which is only slightly lower than the values of state-of-the-art TADF emitters [29,36,37,38].

## 3. Conclusions

The derivatives of pyridazine and 9,9-dimethyl-9,10-dihydroacridine or phenoxazine were obtained by single-step synthesis employing Buchwald–Hartwig cross-coupling reactions. The compounds are characterized by high thermal stabilities. Their 5% weight loss temperatures are 314 and 336 °C. During the TGA measurements, complete weight loss of both pyridazine-based compounds was detected, indicating sublimation rather than thermal degradation of the samples. The derivative of pyridazine and 9,9-dimethyl-9,10-dihydroacridine is capable of glass formation. Its glass transition temperature is 80 °C. The ionization potential values, determined by cyclic voltammetry, are only slightly dependent on the strength of the donor moieties. They are 5.38 and 5.42 eV. The theoretical study revealed that S_0_→S_1_ excitation is characterized by intramolecular charge transfer that is the result of large dihedral angles between the pyridazine moieties and donor fragments in both molecules. The long-living components of the photoluminescence decay curves were detected for the deoxygenated toluene solutions of the compounds, additional to the prompt fluorescence components. The appearance of thermally activated delayed fluorescence (TADF) is presumed from the theoretical analysis of distribution of the highest occupied and lowest unoccupied molecular orbitals and based on the experimental data of time-resolved photoluminescence spectroscopy measurements conducted at different temperatures. The reverse intersystem crossing rate constant of 9.5 × 10^5^ s^−1^ estimated for phenoxazinyl disubstituted pyridazine is higher than that observed for the derivative of 9,9-dimethyl-9,10-dihydroacridine and pyridazine (3.3 × 10^5^ s^−1^), primarily due to the fast TADF.

## Figures and Tables

**Table 1:** Thermal characteristics of the compounds.

Compound	T_ID_, °C ^1^	T_g_, °C	T_cr_, °C ^2^	T_m_, °C ^3^
**2PO-PYD**	314	-	188	248
**2AC-PYD**	336	80	133	231

^1^ Temperature of 5% loss of mass determined by TGA. ^2^ Crystallization temperatures determined by DSC from the first cooling scan. ^3^ Melting points determined by DSC from the second heating scan.

**Table 2:** Photophysical characteristics of **2PO-PYD** and **2AC-PYD**.

Compound	*λ_a_*, nm ^1^	*λ_e_*, nm ^2^	Φ ^3^	E_S1_, eV ^4^	E_T1_, eV ^4^	ΔE_ST_^4^
**2PO-PYD**	308/308	ca. 420, 618/398, 639/565	<0.01	2.68	2.59	0.09
**2AC-PYD**	ca. 280/290	353, 535/346, 561/517	<0.01	2.64	2.29	0.35

^1^ Wavelengths of the bands of absorption spectra of the solutions of the compounds in toluene/THF. ^2^ Wavelengths of the bands of emission spectra of the compounds of the toluene/THF solutions and films. ^3^ PL quantum yield of deoxygenated toluene solutions. ^4^ Derived from spectral data of the films of compounds recorded at 77 K in the absence of oxygen.

**Table 3:** Photophysical characteristics of the toluene solutions of **2PO-PYD** and **2AC-PYD**.

Toluene Solution ^1^	*λ*, nm ^2^	Lifetime, ns ^3^	k_RISC_, s^−1^	k_ISC_, s^−1^	χ ^2,4^
**2PO-PYD**	394	2.85	-	-	1.043
**2PO-PYD**	609	2.49 (85.44%), 9.75 (6.49%), 92.86 (8.07%)	9.5 × 10^5^	2.2 × 10^8^	1.166
**2AC-PYD**	534	6 (95.52%), 142.96 (4.48%)	3.3 × 10^5^	1.6 × 10^8^	1.034

^1^ Deoxygenated. ^2^ Wavelength at which the measurement was performed. ^3^ Intensity amplitude in parentheses. ^4^ Weighted sum of fit points square deviations.

## Data Availability

The data presented in this study are available on request from the corresponding author.

## Supplementary Materials

The following supporting information can be downloaded at: https://www.mdpi.com/article/10.3390/ma16031294/s1, Figure S1: ^1^H spectrum of 2PO-PYD; Figure S2. ^13^C NMR spectrum of **2PO-PYD**; Figure S3. ^1^H spectrum of **2AC-PYD**; Figure S4. ^13^C NMR spectrum of **2AC-PYD**; Figure S5. DSC (a,b) and TGA (c) thermograms of compounds **2PO-PYD**, and **2AC-PYD**; Figure S6. Theoretical UV spectra (in toluene) obtained from TD-DFT calculations of the pyridazine-based compounds **2PO-PYD** (a) and **2AC-PYD** (b); Figure S7. PL decay curves of deoxygenated toluene solutions of **2PO-PYD** (a,b), **2AC-PYD** (c). Refs. [30,31] are cited in the Supporting Information.
