# Supplementary material for: Derivatives of Pyridazine with Phenoxazine and 9,9-Dimethyl-9,10-dihydroacridine Donor Moieties Exhibiting Thermally Activated Delayed Fluorescence
Source: Materials (Basel). 2023 Feb 2;16(3):1294. doi: 10.3390/ma16031294 (PMC9919726; doi:10.3390/ma16031294)
Supplement: Supplementary file 1 [file materials-16-01294-s001.zip › materials-2163712-supplementary.pdf]

Derivatives of Pyridazine with Phenoxazine and 9,9-Dimethyl-9,10-Dihydroacridine Donor Moieties Exhibiting Thermally Activated Delayed Fluorescence

Levani Skhirtladze [1], Oleksandr Bezvikonnyi [1,2], Rasa Keruckienė [1], Lukas Dvylys [1], Malek Mahmoudi [1], Linas Labanauskas [3], Azhar Ariffin [4] and Juozas V. Grazulevicius [1,*]

1 Department of Polymer Chemistry and Technology, Faculty of Chemical Technology, Kaunas University of Technology, LT-51423 Kaunas, Lithuania
2 Department of Physics, Faculty of Mathematics and Natural Science, Kaunas University of Technology, LT-51369 Kaunas, Lithuania
3 Center for Physical Sciences & Technology, Department of Organic Chemistry, LT-10257 Vilnius, Lithuania
4 Department of Chemistry, Faculty of Science, Universiti Malaya, Kuala Lumpur 50603, Malaysia
* Correspondence: juozas.grazulevicius@ktu.lt

Table of Contents:

Experimental Section 2
Figures 4
References 10

Experimental Section

Instrumentation

^{1}H NMR and ^{13}C NMR spectra were recorded with a Bruker Avance III apparatus (400 and 101 MHz). The samples were prepared by dissolving 15 mg of the material in 0.7 mL of deuterated chloroform ($CDCl_3$). The data are presented as chemical shifts (δ) in ppm (in parentheses: multiplicity, coupling constant, and integration).

IR spectra were recorded with a Vertex 70 Bruker spectrometer equipped with an ATR attachment with a diamond crystal over frequencies of 600–3500 cm^{-1} with a resolution of 5 cm^{-1} over 32 scans. The IR spectra were presented as a function of transparency (T) expressed in percent (%) against the wavenumber (v) expressed in cm^{-1}.

Mass (MS) spectra were recorded on a Waters ZQ 2000 mass spectrometer.

Cyclic voltammetry measurements were performed by using a glassy carbon working electrode (a disk with the diameter of 2 mm) in a three-electrode cell of Autolab Type potentiostat – galvanostat. The measurements were carried out for the solutions in dry dichloromethane containing 0.1 M tetrabutylammonium hexafluorophosphate at 25 °C; the scan rate was 50 mV/s while the sample concentration was 10–3 M. The potentials were measured against silver as a quasi-reference electrode. Platinum wire was used as a counter electrode. The potentials were calibrated with the standard ferrocene/ferrocenium (Fc/Fc+) redox system.

Differential scanning calorimetry (DSC) measurements were carried out with a TA Instruments Q2000 calorimeter. The samples were heated and cooled at a scan rate of 10°C/min under nitrogen atmosphere. Thermogravimetric analysis (TGA) was performed with TA Instruments Q50 analyser. The samples were heated at a rate of 20 °C/min.

Theoretical calculations. The ground-state geometries were optimized by using the B3LYP (Becke three parameters hybrid functional with Lee-Yang-Perdew correlation) [30]functional at 6-31G (d, p) level in vacuum with the Gaussian[[31] program.

Firstly, the equilibrium conformer search at the ground state was performed by using the MMFF (Molecular mechanics force fields) method, and then this geometry was used for further optimization. The vertical singlet and triplet energy values were calculated by using the energy values at the corresponding excited state geometry. The time-dependent DFT (TD-DFT) calculations were carried out with the Gaussian 16 software package. Molecular orbitals were visualized by using Gaussview.

UV-visible absorption spectra of dilute 10^{-5} M solutions and neat films were recorded with an UV–VIS–NIR Avantes (AvaSpec-2048XL) spectrophotometer. PL spectra of 10^{-5} M solutions, solid mixture and dopant free films of the compounds were recorded at different temperatures by using of Edinburgh Instruments' FLS980 Fluorescence Spectrometer. The integrated sphere was used to obtain Φ of the deoxygenated toluene solutions by an absolute method. Photoluminescence decay curves of the deoxygenated toluene solutions of the compounds were recorded with the Edinburgh Instruments FLS980 spectrometer and PicoQuant LDH-D-C-375 laser (wavelength 374 nm) as the excitation source. The Optistat DN2 cryostat and turbomolecular pump were used for recording the spectra at different temperatures. FLS980 software was used for an estimation of lifetimes and yields.

Materials and methods

All reagents and chemicals were purchased and used without additional purification. Solvents were distilled and deaerated before use. All reactions were carried out under inert atmospheres (nitrogen or argon) using standard Schlenk techniques. Fluka Silica Gel 60 was used for column chromatography and thin layer chromatography (TLC).

Materials used in this work include the 3,6-Dibromopyridazine (purity 98.0%), 10H-Phenoxazine (purity 98.0%), 9,9-dimethyl-9,10-dihydroacridine (purity 95.0%), Tris(dibenzylideneacetone)dipalladium (0) (purity 99.0%), 2-(Dicyclohexylphosphino)-2',4',6'-tri-isopropyl-1,1'-biphenyl (purity 98.0%) (all purchased from Fluorochem), Sodium tert-butoxide (purity 97.0%) (purchased from Sigma-Aldrich).

3,6-di(10H-phenoxazin-10-yl)pyridazine (2PO-PYD) A mixture of 3,6-dibromopyridazine (0.5g, 2.1mmol), 10H-phenoxazine (0.84g, 4.58mmol), tris(dibenzylideneacetone) dipalladium (0) (0,096g, 0.104mmol), X-Phos (0.07g, 0.146mmol), sodium tert-butoxide (0.44g, 4.57mmol), dry toluene (10mL) were added in a flask under argon atmosphere. The reaction mixture was stirred at 110–120°C for 24 hours. When the reaction was completed, crude product was purified by column chromatography by using silica, EA/Hex=1/4 as an eluent. The target compound was obtained as yellow crystals. Yield: 0.203g (21.8%). T_m=248 ºC (DSC).

^{1}H NMR (400 MHz, $CDCl_3$), δ 7.47 (s, 2H), 7.15 (d, J = 7.5 Hz, 4H), 7.04 – 6.86 (m, 12H).

^{13}C NMR (101 MHz, $CDCl_3$), δ 155.6, 148.2, 131.8, 124.8, 124.6, 123.7, 119.5, 116.8.

FT-IR (KBr): 3055 (Ar C-H), 2953, 2932 (Alk C-H), 1489, 1420 (Ar C=C), 1326, 1274 (Ar C-N), 793 (Alk C-H).

Molecular formula $C_{28}H_{18}N_4O_2$, calculated m/z = 442.14, observed m/z =443.05.

3,6-bis(9,9-dimethylacridin-10(9H)-yl)pyridazine (2AC-PYD) A mixture of 3,6-dibromopyridazine (0.5g, 2.1mmol), 9,10-dihydro-9,9-dimethylacridine (0.96g, 4.30mmol), tris(dibenzylideneacetone) dipalladium (0) (0,096g, 0.104mmol), X-Phos (0.07g, 0.146mmol), sodium tert-butoxide (0.44g, 4.57 mmol), dry toluene (10mL) were added in a flask under argon atmosphere. The reaction mixture was stirred at 110–120°C for 24 hours. When the reaction was completed, crude product was purified by column chromatography by using silica, Dcm/Hex=1/4 as an eluent. The target compound was obtained as yellow crystals. Yield: 0.067g (6.4%). T_m=231 ºC (DSC).

^{1}H NMR (400 MHz, $CDCl_3$), δ 7.50 (d, J = 7.6 Hz, 4H), 7.39 (d, J = 6.0 Hz, 6H), 7.20 (t, J = 7.1 Hz, 4H), 7.13 (t, J = 7.1 Hz, 4H), 1.65 (s, 12H).

^{13}C NMR (101 MHz, $CDCl_3$), δ 155.7, 140.1, 137.2, 126.6, 124.5, 123.9, 120.3, 37.2, 28.9.

FT-IR (KBr): 3044 (Ar C-H), 2974, 296, 2952 (Alk C-H), 1592, 1475, 1418 (Ar C=C), 1323, 1271 (Ar C-N), 743 (Alk C-H).

Molecular formula $C_{34}H_{30}N_4$, calculated m/z = 494.25, observed m/z = 495.20.

Figures

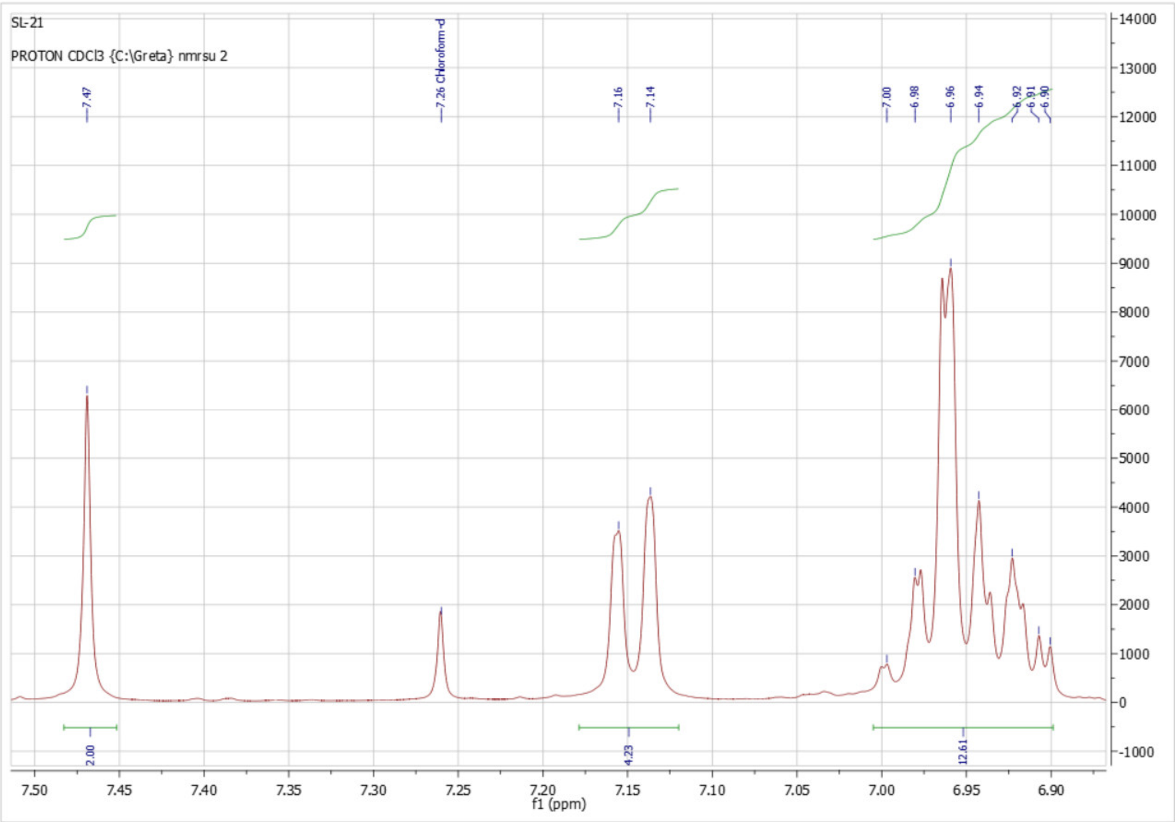

Figure S1. ^{1}H spectrum of **2PO-PYD**.

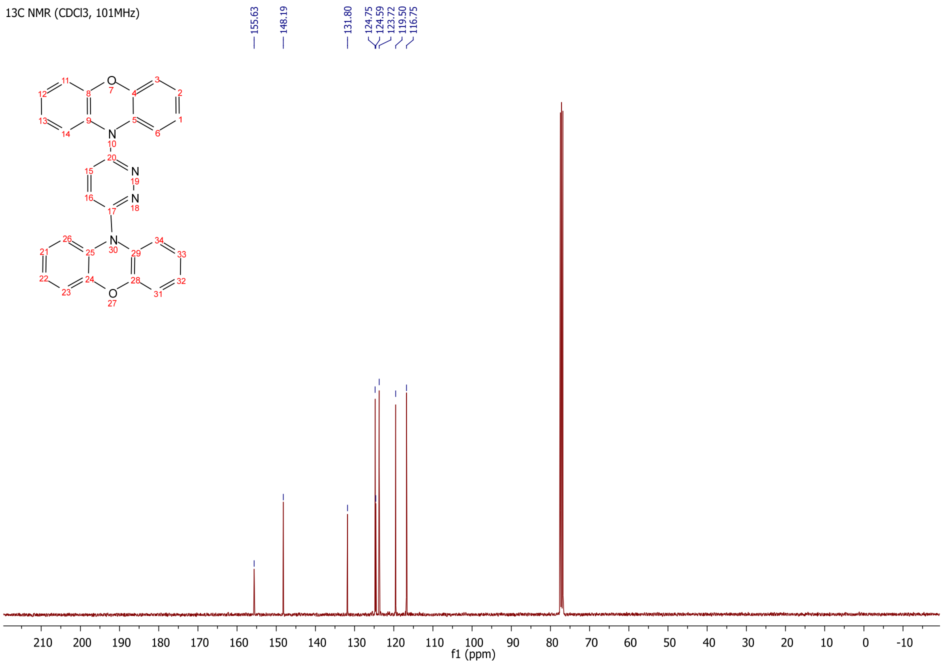

Figure S2. ^{13}C NMR spectrum of **2PO-PYD**.

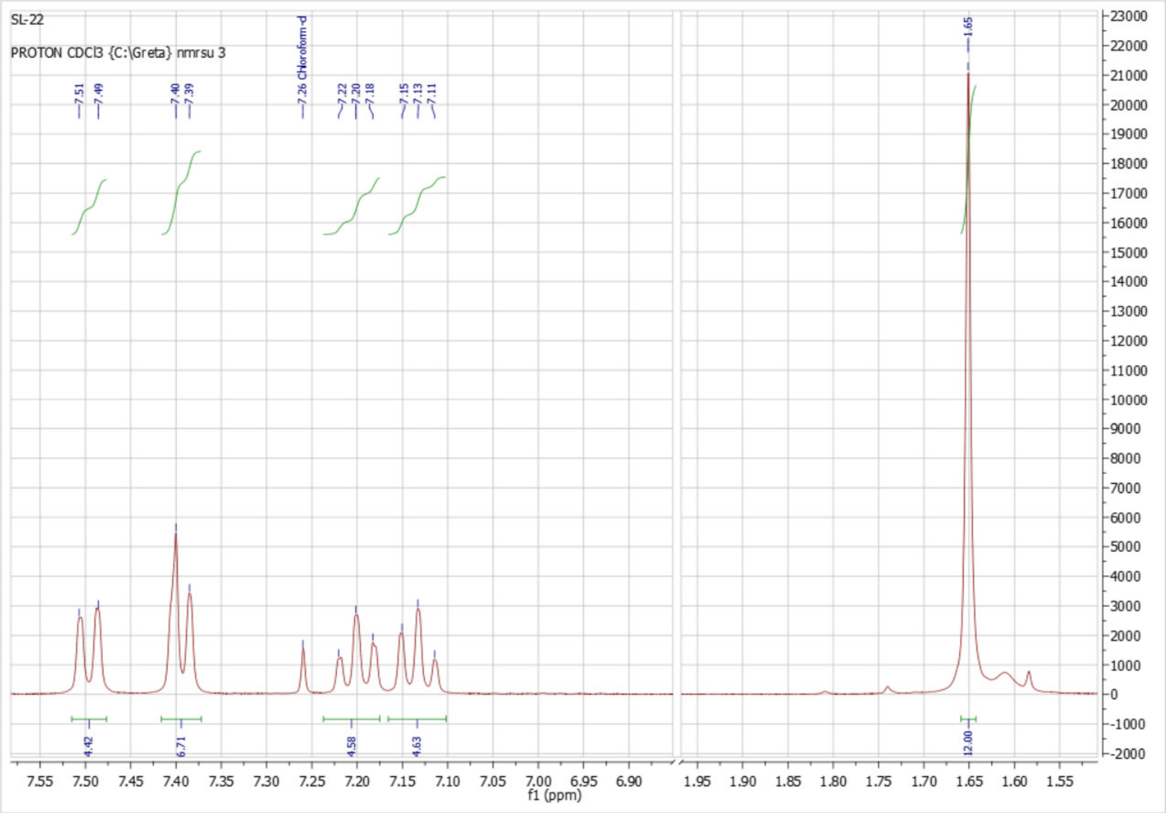

Figure S3. ^{1}H spectrum of **2AC-PYD**.

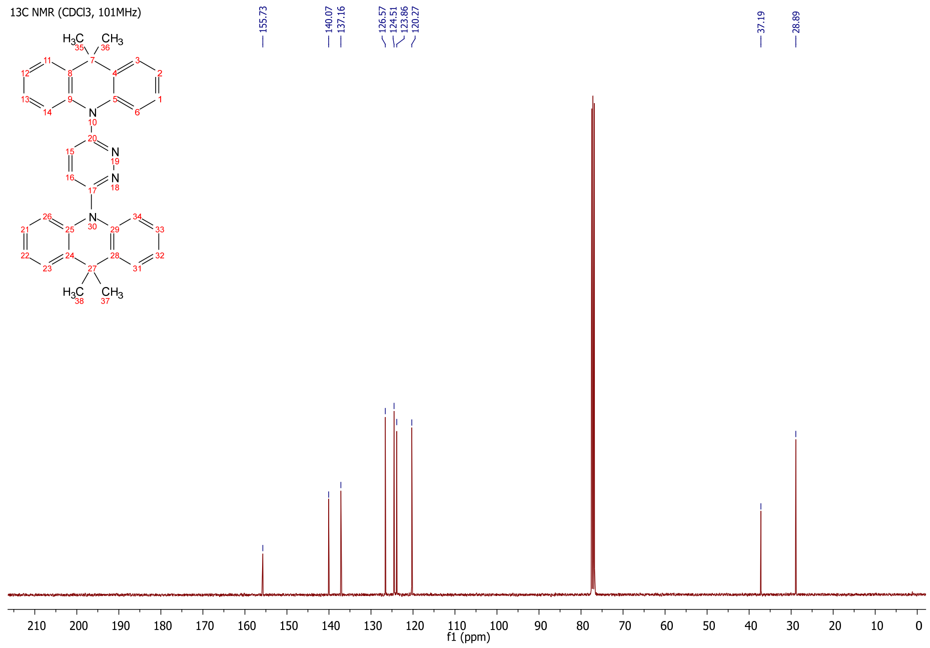

Figure S4. ^{13}C NMR spectrum of **2AC-PYD**.

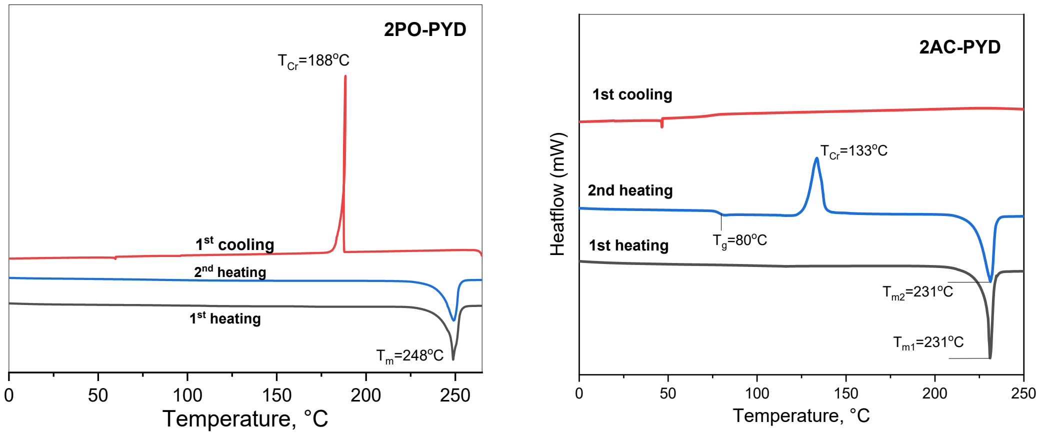

a b

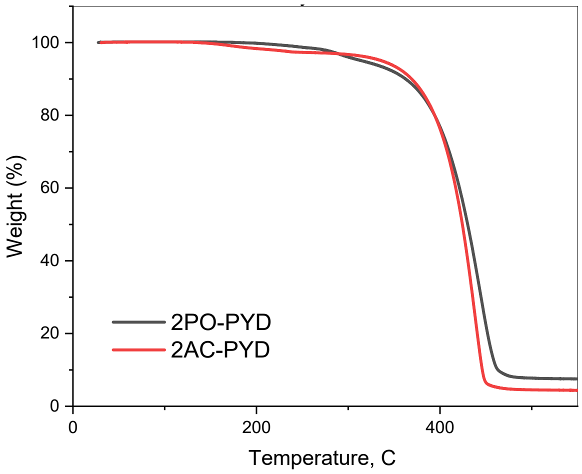

c

Figure S5. DSC (a, b) and TGA (c) thermograms of compounds **2PO-PYD**, and **2AC-PYD**.

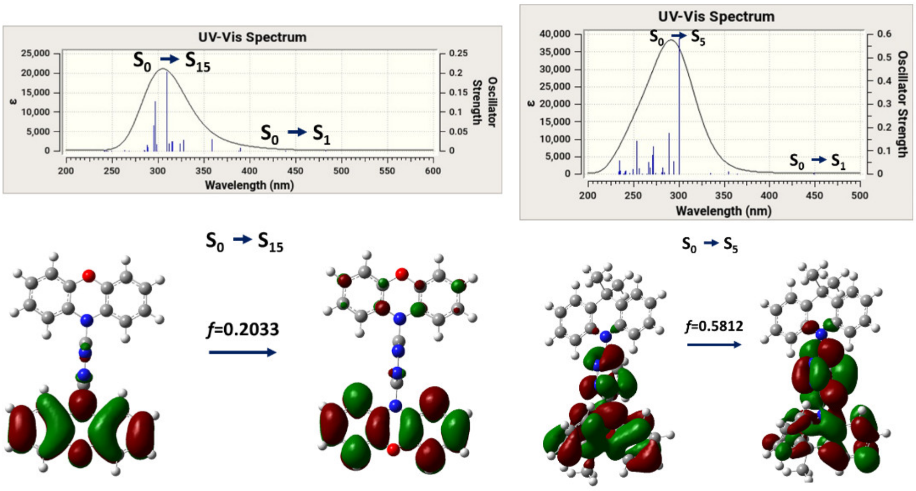

a b

Figure S6. Theoretical UV spectra (in toluene) obtained from TD-DFT calculations of pyridazine-based compounds **2PO-PYD** (a) and **2AC-PYD** (b).

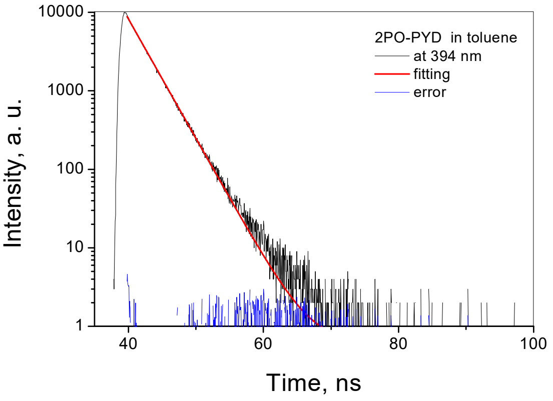

a

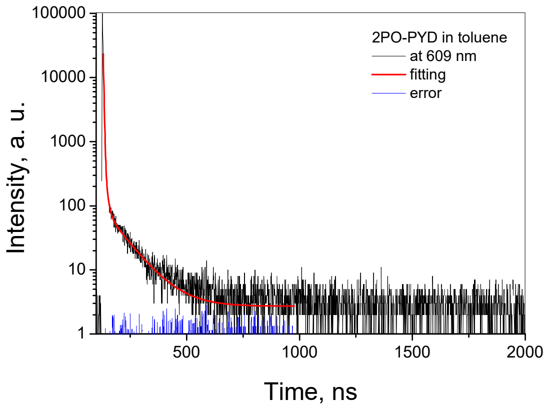

b

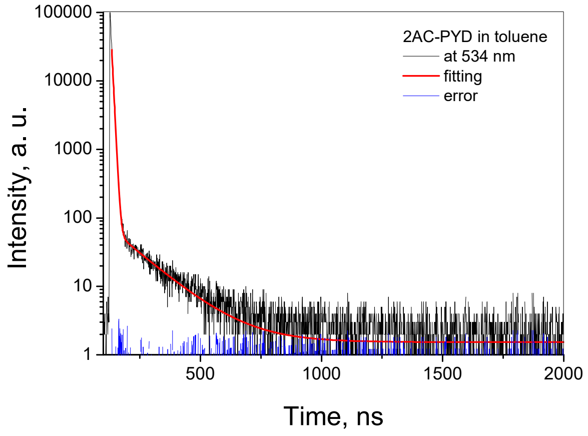

c

Figure S7. PL decay curves of deoxygenated toluene solutions of **2PO-PYD** (a, b), **2AC-PYD** (c).

References

[30] Becke, A.D. Density-functional exchange-energy approximation with correct asymptotic behavior, *Phys. Rev. A.* **1988**, *38*, 3098–3100. https://doi.org/10.1103/PhysRevA.38.3098.

[31] M.J. Frisch, G.W. Trucks, H.B. Schlegel, G.E. Scuseria, M.A. Robb, J.R. Cheeseman, G. Scalmani, V. Barone, G.A. Petersson, H. Nakatsuji, et. al, Gaussian 09, Revision A.02, Gaussian, Inc. Wallingford CT. (2016).
